# Novel Blood Vascular Endothelial Subtype-Specific Markers in Human Skin Unearthed by Single-Cell Transcriptomic Profiling

**DOI:** 10.3390/cells11071111

**Published:** 2022-03-25

**Authors:** Yuliang He, Carlotta Tacconi, Lothar C. Dieterich, Jihye Kim, Gaetana Restivo, Epameinondas Gousopoulos, Nicole Lindenblatt, Mitchell P. Levesque, Manfred Claassen, Michael Detmar

**Affiliations:** 1 Institute of Pharmaceutical Sciences, Swiss Federal Institute of Technology (ETH) Zürich, 8093 Zürich, Switzerland; yuliang.he@pharma.ethz.ch (Y.H.); carlotta.tacconi@unimi.it (C.T.); lothar.dieterich@pharma.ethz.ch (L.C.D.); jihye.kim@pharma.ethz.ch (J.K.); 2 Department of Biosciences, University of Milan, 20133 Milan, Italy; 3 Department of Dermatology, University Hospital Zürich, 8091 Zürich, Switzerland; gaetana.restivo@usz.ch (G.R.); mitchell.levesque@usz.ch (M.P.L.); 4 Department of Plastic Surgery and Hand Surgery, University Hospital Zürich, 8091 Zürich, Switzerland; epameinondas.gousopoulos@usz.ch (E.G.); nicole.lindenblatt@usz.ch (N.L.); 5 Department of Internal Medicine I, University of Tübingen, 72074 Tübingen, Germany; manfred.claassen@med.uni-tuebingen.de; 6 Department of Computer Science, University of Tübingen, 72074 Tübingen, Germany

**Keywords:** blood vascular endothelial cells, human skin, vascular heterogeneity, single-cell RNA sequencing

## Abstract

Ample evidence pinpoints the phenotypic diversity of blood vessels (BVs) and site-specific functions of their lining endothelial cells (ECs). We harnessed single-cell RNA sequencing (scRNA-seq) to dissect the molecular heterogeneity of blood vascular endothelial cells (BECs) in healthy adult human skin and identified six different subpopulations, signifying arterioles, post-arterial capillaries, pre-venular capillaries, post-capillary venules, venules and collecting venules. Individual BEC subtypes exhibited distinctive transcriptomic landscapes associated with diverse biological pathways. These functionally distinct dermal BV segments were characterized by their unique compositions of conventional and novel markers (e.g., arteriole marker GJA5; arteriole capillary markers ASS1 and S100A4; pre-venular capillary markers SOX17 and PLAUR; venular markers EGR2 and LRG1), many of which have been implicated in vascular remodeling upon inflammatory responses. Immunofluorescence staining of human skin sections and whole-mount skin blocks confirmed the discrete expression of these markers along the blood vascular tree in situ, further corroborating BEC heterogeneity in human skin. Overall, our study molecularly refines individual BV compartments, whilst the identification of novel subtype-specific signatures provides more insights for future studies dissecting the responses of distinct vessel segments under pathological conditions.

## 1. Introduction

Blood vessels (BVs) play a pivotal role in the maintenance of tissue homeostasis. They are responsible for the delivery of oxygen and nutrients, as well as the disposal of metabolic waste from peripheral tissues. The blood vasculature possesses a hierarchical structure, where large arteries extend towards smaller arterioles that further ramify into the capillary plexus and eventually converge into venules and larger veins, constituting a closed blood circulatory system [1]. Endothelial cells (ECs) that form the inner lining of BVs exhibit site-specific differences in their morphological and functional properties. Whilst the vascular tone is primarily regulated at the level of arterioles, capillaries constitute the major surface for gas and fluid exchange and capillary ECs are interconnected by tight and adherence junctions [2]. In contrast, post-capillary venular ECs lack tight junctions, rendering them more intrinsically leaky compared to arterioles and continuous capillaries [3]. BVs undergo dramatic remodeling under pathological conditions, including vascular enlargement and hyperpermeability. Inflammatory mediators induce the upregulation or de novo expression of adhesion molecules selectively on venular ECs, which enables the tethering, adhesion and subsequent extravasation of circulating leukocytes through venules and generally not adjacent capillaries [4]. Intriguingly, it has previously been shown that existing capillaries may also adopt venule-like phenotypes in response to inflammatory stimuli [5].

Extensive efforts to unravel the lineage-specific differentiation of blood vascular endothelial cells (BECs) during embryonic development have furthered our understanding of the molecular patterning of arteriovenous specification. Of note, BECs lining different vessel compartments or derived from different tissues manifest diverse transcriptomic and epigenomic landscapes that govern their lineage specificity and enable organ-specific crosstalk with immune cells [6,7,8]. Recent advances in single-cell transcriptomic profiling technologies have provided more insights into the intercellular heterogeneity among different EC subpopulations in a multitude of murine organs [9,10,11,12]. In particular, Vanlandewijck et al. decoded specific molecular signatures of ECs along the arteriovenous axis in adult mouse brain and illuminated gradual transcriptomic changes that transit from arteries along capillaries towards the veins [13].

The skin serves as a physical and immunological barrier to the outer environment, where functionally distinct BV compartments play different roles under homeostatic conditions and in inflammatory skin diseases. Many studies have collectively surveyed the heterogeneous cell populations present in healthy human skin, including keratinocytes, fibroblasts and myeloid cells [14,15,16,17]. To broaden our knowledge of BEC diversity and their spatial organization in human skin, we performed single-cell RNA sequencing (scRNA-seq) in conjunction with large-scale immunofluorescence staining to finely delineate blood vascular endothelial heterogeneity. Our findings provide an in-depth characterization of distinct BEC subtypes and uncover novel markers discerning the vascular zonation in healthy adult human skin.

## 2. Materials and Methods

### 2.1. Isolation of Human Dermal Endothelial Cells

Healthy skin biopsies of breast tissue from 2 different donors were obtained from the plastic surgery department of the University Hospital Zürich (USZ, Zürich, Switzerland) with the assistance of the biobank of the dermatology department (KEK n° 647) and the support of SKINTEGRITY.CH. Only biopsies from donors who gave a written consent were used for further analyses. The use of human samples was approved by the ethic commission of canton Zürich (KEK n° 2017-00687). Skin tissues were first washed with Hank’s Balanced Salt Solution (HBSS) supplemented with 5% FBS (Gibco, Waltham, MA, USA), 2% antibiotic and antimycotic solution (AA, Gibco) and 20 mM HEPES (Gibco) before incubating with the dermal side down in 0.25% trypsin (Sigma-Aldrich, Burlington, MA, USA) at 4 °C overnight. Trypsin digestion was quenched by washing with RPMI basal medium supplemented with 10% FBS, 2% AA, and 20 mM HEPES. The epidermal sheets were separated from the dermis, which was subsequently minced and digested enzymatically (1000 units/mL Collagenase type 1 (Worthington, Columbus, OH, USA), 40 µg/mL DNase (Roche, Basel, Switzerland) in RPMI basal medium supplemented with 10% FBS, 2% AA, and 20 mM HEPES) at 37 °C for 1 h under constant agitation. Digested skin tissues were filtered through a cell strainer (100 µm; Falcon, Glendale, AZ, USA), further washed with RPMI basal medium and centrifuged at 1500 rpm for 6 min at 4 °C. The cell suspension was washed with FACS buffer (DPBS with 2% FBS and 1 mM EDTA) and labeled with FITC-conjugated mouse anti-human CD45 (1:25, clone HI30, 304006, BioLegend, San Diego, CA, USA), PE-conjugated mouse anti-human CD31 (1:25, clone WM59, 555446, BD Biosciences, Franklin Lakes, NJ, USA) and Zombie-NIR (1:500, 423106, BioLegend) for 30 min at 4 °C. Single living CD45-CD31+ endothelial cells were filtered through a 40 µm cell strainer (Falcon) and sorted directly into 384-well plates containing 0.8 µL of lysis buffer (0.1% Triton X-100, 2.5 mM dNTPs, 2.5 µM oligo-dT, 1 units/µL RNasin Plus RNase inhibitor (Promega, Madison, WI, USA)) using a FACSAria II Flow Cytometer (BD Biosciences) with a 100 µm nozzle as previously described [18]. Plates were immediately centrifuged and stored at −80 °C before further processing.

### 2.2. Full-Length Single-Cell RNA Sequencing

Library preparation and sequencing steps were performed at the Functional Genomic Center Zürich (FGCZ, Zürich, Switzerland). In brief, cDNA libraries were prepared in accordance with a miniaturized version of the Smart-seq2 protocol [19] using the Mosquito HV pipetting robot (TTP Labtech, Melbourn, UK). Reverse transcription in a final volume of 2 µL and cDNA amplification in a final volume of 5 µL were performed, after which cDNA quality was assessed by a 2100 Bioanalyzer (Agilent Technologies, Santa Clara, CA, USA). cDNA (0.1 ng) derived from single cells was individually tagmented using the Nextera XT kit (Illumina, San Diego, CA, USA) in a final volume of 5 µL, followed by barcoding and library amplification in a final volume of 10 µL. The resulting 384 libraries were subsequently pooled, double-sided size selected (0.5× followed by 0.8× ratio using Ampure XP beads (Beckman Coulter, Brea, CA, USA)) and measured by the 4200 TapeStation System (Agilent Technologies, Santa Clara, CA, USA). The pooled libraries were sequenced in Illumina HiSeq 4000 with single-read 125 bp chemistry and on average around 750,000 reads per cell.

### 2.3. Preprocessing of scRNA-Seq Data

Low quality bases and the Nextera adapter sequences were removed using trimmomatic v0.33 [20]. Trimmed reads were aligned to the Ensembl homo sapiens genome assembly GRCh38 (release 92) using STAR v2.4.2a [21]. Gene expression was quantified with the ‘featureCounts’ function in the Rsubread package v1.26.1 [22]. Quality filtering was performed with the scran package v1.4.5, where cells with a library size or feature size 2.5 median absolute deviations (MADs) outside of the median, or with mitochondrial contents 3 MADs above the median were removed as outliers [23]. Genes expressed in at least 10% of cells were grouped on the basis of their count-depth relationship by SCnorm v0.99.7 that applied a quantile regression within each group to estimate scaling factors and subsequently normalize for sequencing depth [24]. Cells with CD45 positivity were also removed before downstream analyses.

### 2.4. Clustering, Differential Expression and Pseudotime Trajectory Analyses

To accommodate batch effects across different sample donors, we matched mutual nearest neighbors (MNNs) using the ‘fastMNN’ function implemented in the Batchelor package v1.0.1 [25]. Gene-specific variance was decomposed into biological and technical components. The genes displaying positive biological variances (FDR < 0.05, mean expression level between 0.25 and 4) were defined as variable features, which were then subjected to merged principal component analysis (PCA) across all batches. Unsupervised clustering was performed on the corrected low-dimensional coordinates (PC 1–10) using the Seurat package v2.3.4 [26] and visualized with Uniform Manifold Approximation and Projection (UMAP) [27]. Differentially expressed genes among the clusters were defined by the ‘FindMarkers’ function (min.pct = 0.20, logfc.threshold = 0.25, p_val_adj < 0.05) using the MAST test [28]. Reactome pathway analysis was performed on marker genes of each cluster to identify enriched gene ontology with the ClusterProfiler package v3.14.0 [29]. The expression patterns of selected markers were plotted by the ‘FeaturePlot’ function using the corrected expression values. For pseudotime trajectory analysis, the Seurat outputs were first loaded as a lineage tree object, filtered genes (minexpr = 90, minnumber = 100) were used to generate the distance matrix and the StemID2 algorithm was applied to compute the entropy of each cell and calculate cell projections. A lineage graph could be generated based on the pseudotime trajectories predicted by StemID2, and the average pseudo-temporal expression profiles of our selected markers were visualized with the ‘plotexpression’ function [30]. All analyses of scRNA-seq data were performed with R v3.4.0.

### 2.5. Immunofluorescence Staining of Skin Sections

Healthy skin samples from breast and abdomen were obtained from consenting adult donors and used after approval by the ethic commission of canton Zürich (KEK n° 2017-00687). Samples were embedded in OCT compound and snap-frozen on dry ice prior to cryostat sectioning (7 μm). After fixation in 4% paraformaldehyde (PFA), skin sections were incubated in blocking solution (5% donkey serum, 0.2% BSA, 0.3% Triton X-100 and 0.05% NaN_3_ in PBS) for 1 h. Subsequently, the sections were incubated at 4 °C overnight with the respective primary antibodies, which include rabbit anti-human LYVE-1 (1:100, 102PA50AG, ReliaTech, Wolfenbüttel, Germany), biotinylated goat anti-human LYVE-1 (1:100, BAF2089, R&D, Minneapolis, MN, USA), mouse anti-human CD31 (1:100, clone JC70A, M0823, Dako, Santa Clara, CA, USA), rabbit anti-human CD31 (1:40, GTX110602, GeneTex, Irvine, CA, USA), rabbit anti-human ERG (1:100, ab92513, Abcam, Cambridge, UK), goat anti-human ERG (1:50, NB100-2472, Novus, Centennial, CO, USA), goat anti-human ACKR1 (1:200, NB100-2421, Novus), mouse anti-human ICAM1 (1:100, clone W-CAM-1, MA5-13021, Invitrogen, Waltham, MA, USA), sheep anti-human ICAM1 (1:100, AF720, R&D), rabbit anti-human HEY1 (1:100, ab22614, Abcam), mouse anti-human SELP (1:300, clone AK-6, ab6632, Abcam), goat anti-human EFNB2 (1:100, AF496, R&D), rabbit anti-human EGR2 (1:200, ab43020, Abcam), rabbit anti-human LRG1 (1:200, HPA001888, Atlas Antibodies, Bromma, Sweden), mouse anti-human GJA5 (1:250, clone 2F9A11, 37-8900, Invitrogen), mouse anti-human ASS1 (1:100, clone 2B10, ab124465, Abcam), rabbit anti-human S100A4 (1:200, ab41532, Abcam), goat anti-human SOX17 (1:10, AF1924, R&D) and mouse anti-human PLAUR (1:100, clone 62022, MAB807, R&D). Following washing, skin sections were incubated at room temperature for 30 min with the corresponding secondary antibodies conjugated with Alexa488, Alexa594 or Alexa647 (donkey anti-mouse, donkey anti-rabbit, donkey anti-goat, donkey anti-sheep; Thermo Fisher, Waltham, MA, USA) together with Hoechst 33342 (1:1000, Sigma-Aldrich) for nuclear counterstaining. Images were acquired with an LSM 780 upright confocal microscope (Zeiss, Jena, Germany) and analyzed with Fiji [31]. The goat anti-human ERG antibody also stained unspecifically the epidermis of healthy human skin.

### 2.6. Whole-Mount Immunostaining

Skin blocks were fixed with 4% PFA at 4 °C overnight, permeabilized in 1% Triton X-100 and 0.5% Tween-20 in PBS for 2 days, blocked in Perm/Block Solution (1% BSA, 0.1% Tween-20 and 0.03% NaN_3_ in PBS) for 3 days and stained at 4 °C for 7 days with the respective primary antibodies: rabbit anti-human ERG (1:100), goat anti-human ERG (1:00), goat anti-human ACKR1 (1:200), mouse anti-human ICAM1 (1:100), sheep anti-human ICAM1 (1:100), rabbit anti-human HEY1 (1:100), mouse anti-human SELP (1:300), goat anti-human EFNB2 (1:100), rabbit anti-human EGR2 (1:200), mouse anti-human ASS1 (1:100), goat anti-human SOX17 (1:50), mouse anti-human PLAUR (1:100), followed by washes and incubation with the corresponding secondary antibodies at 4 °C for 7 days. Optical clearing was performed as previously described [32]. Skin blocks were embedded in 1% ultrapure LMP Agarose (Thermo Fisher) after staining and dehydrated sequentially in 50%, 70%, 95% and 100% methanol. Pre-clearing in 50% BABB (benzyl alcohol/ benzyl benzoate 1:2) in methanol was followed by clearing in 100% BABB overnight. Whole-mount images were acquired with an LSM 880 inverted confocal microscope (Zeiss) and analyzed with Fiji.

## 3. Results

### 3.1. Identification of Six Distinct Human Dermal Blood Vascular Endothelial Cell Subtypes

**Figure 1:**
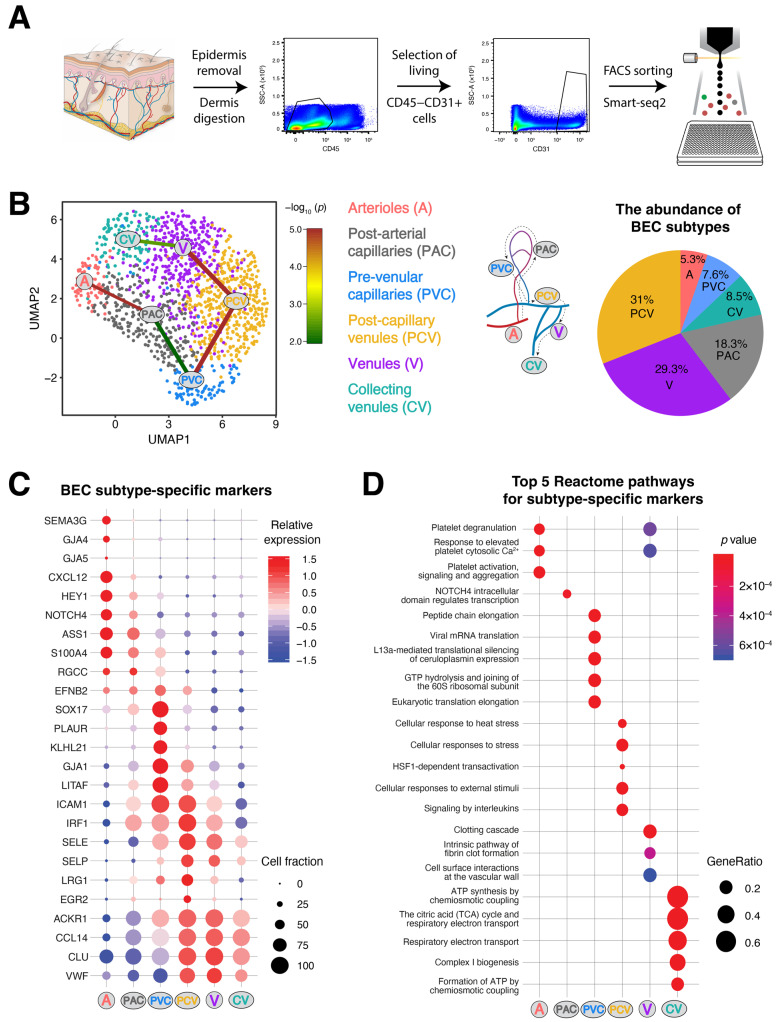
Distinct transcriptomic patterns signify six human dermal blood vascular endothelial cell subtypes. (**A**) Isolation workflow of endothelial cells (ECs) from healthy human skin tissues for scRNA-seq. (**B**) Identification of six different subtypes of BECs, with each cluster corresponding to a particular vessel segment as denoted in the middle panel: arterioles (A), post-arterial capillaries (PAC), pre-venular capillaries (PVC), post-capillary venules (PCV), venules (V) and collecting venules (CV). The connections among different clusters indicate the inferred pseudotime trajectories (*p* < 0.05) using the StemID2 algorithm, where the color of the connections denotes the −log_10_ (*p* value) and the thickness reflects how densely a connection is populated with cells. The abundance of each BEC subtype within the total population is shown in the right panel. (**C**) Expression patterns of selected markers in different BEC clusters, where color intensity indicates the relative abundance level and the dot size denotes the proportion of cells with detectable expression in the respective cluster. (**D**) Top 5 Reactome pathways enriched in each BEC subpopulation. ‘GeneRatio’ indicates the ratio of genes from our input list that are related to a specific pathway over the total number of genes used as input.

To investigate the heterogeneity among different subtypes of dermal BVs, we isolated single living CD45− stromal cells, enriching for the CD31+ endothelial population from healthy human skin tissues (Table S1) for single-cell transcriptomic profiling (1536 cells) using the Smart-seq2 approach (Figure 1A). The sorted cells of low quality and *CD45* positivity were removed prior to the downstream analysis. While all the ECs exhibited a comparable level of platelet and endothelial cell adhesion molecule 1 (*PECAM1*, also known as *CD31*) expression, we identified two dissimilar populations of ECs from unsupervised clustering, namely the BECs (1335 cells) and lymphatic endothelial cells (LECs; 39 cells) as signified by the expression of plasmalemma vesicle-associated protein (*PLVAP*) and lymphatic vessel endothelial hyaluronan receptor 1 (*LYVE1*), respectively (Figure S1A). Owing to the paucity of LECs in the dataset, we focused our attention on the BEC cluster for further analyses. From unsupervised clustering, we identified six individual subtypes of BECs, ranging from arterioles (A), post-arterial capillaries (PAC), pre-venular capillaries (PVC), post-capillary venules (PCV), venules (V) to collecting venules (CV), in accordance with their transcriptomic signatures (Figure 1B,C and Figure S1B,C). The majority of the BEC population was comprised of capillaries and venules, whereas the arterioles merely constituted around 5% of the cells. Nonetheless, it is evident that different BEC subtypes manifest distinctive transcriptomic profiles when compared among each other (Table S2). In line with the literature, genes related to the Notch signaling pathway (*HEY1* and *NOTCH4*) were most enriched in the arteriole and PAC clusters [33]. In contrast, we observed higher expression levels of adhesion molecules, such as E-selectin (*SELE*), P-selectin (*SELP*) and intercellular adhesion molecule 1 (*ICAM1*), in the venular compartments (Figure 1C) [4]. Of note, the level of ephrin B2 (*EFNB2*) was found to be more abundant in the pre-venular vessels, as opposed to the expression pattern of the von Willebrand factor (*VWF*). Intriguingly, pseudo-temporal reconstruction inferred a trajectory connecting these BEC compartments, which reflects the nature of BVs transporting along a continuum (Figure 1B). In addition, we also identified a list of transcription factors that are differentially expressed among the BEC subtypes (Figure S1D). Gene ontology analysis highlighted pathways associated with the markers upregulated in each cluster, as exemplified by NOTCH4 signaling in the PAC cluster, cellular stress responses and interleukin signaling in the PCV cluster, vascular wall cell surface interactions in the V cluster and ATP synthesis in the CV cluster (Figure 1D).

### 3.2. Discrimination between Venular and Non-Venular Endothelial Cells via the Expression of Established Markers

**Figure 2:**
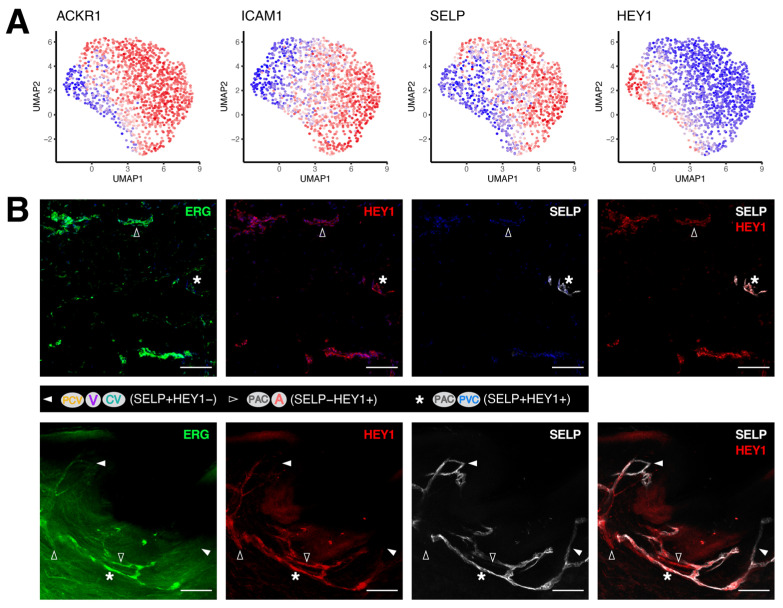
Venular and non-venular endothelial cells in adult human skin can be distinguished by conventional markers. (**A**) Expression patterns of the venular marker ACKR1, the adhesion molecules ICAM1 and SELP and the arterial marker HEY1. (**B**) Representative section (upper) and whole-mount staining (lower) of the pan-endothelial marker ERG (green), HEY1 (red) and SELP (white) distinguish individual BV compartments. White arrowheads, empty arrowheads and asterisks indicate the SELP+HEY1−, SELP−HEY1+ and SELP+HEY1+ populations, respectively. Scale bars: 100 µm.

It has recently been shown that the atypical chemokine receptor 1 (ACKR1) could distinguish venular ECs from non-venular ECs in murine skin [34]. We observed a similar pattern in human skin, where *ACKR1* expression was primarily found in the venular compartment, contrary to that of *HEY1* (Figure 2A and Figure S2A). While the expression of *ICAM1* and *SELP* largely resembled the pattern of *ACKR1*, distinguishable differences were found in the PAC and CV clusters. To validate marker expression at the protein level and marker co-localization at individual BV segments, we performed immunofluorescence staining on skin sections derived from at least three other healthy donors. In combination with the pan-endothelial marker CD31 and the LEC marker LYVE1 for denoting lymphatic vessels (LVs), we observed that positive staining of ACKR1, ICAM1 or HEY1 was solely found in CD31+LYVE1− dermal BVs (3/3 donors, Figure S2B–D). To further examine the variety of BEC subtypes in human skin, we co-stained the arterial marker HEY1 with the pan-endothelial marker ERG [35] and SELP or ICAM1 (Figure 2B and Figure S2E). In consistence with the expression patterns that we observed in the scRNA-seq data, we were able to identify distinct BV subtypes corresponding to our BEC clusters, as exemplified by ICAM1−HEY1+ arteriole-capillary segments in contrast to ICAM1+HEY1− venular vessels (3/3 donors, Figure S2E). Additionally, we gained a more detailed view of the spatial expression of HEY1 and SELP along the BVs using whole-mount staining (Figure 2B). While SELP−HEY1+ vessels matched arterioles and the lower fraction of the PAC cluster, their partial overlap in the PVC cluster and the upper part of the PAC cluster was denoted by BVs positive for both markers. On the contrary, the venular ECs that include the PCV, V and CV clusters were devoid of HEY1 expression (4/4 donors).

### 3.3. Unique Marker Combinations Delineate Venular Endothelial Cell Subpopulations

**Figure 3:**
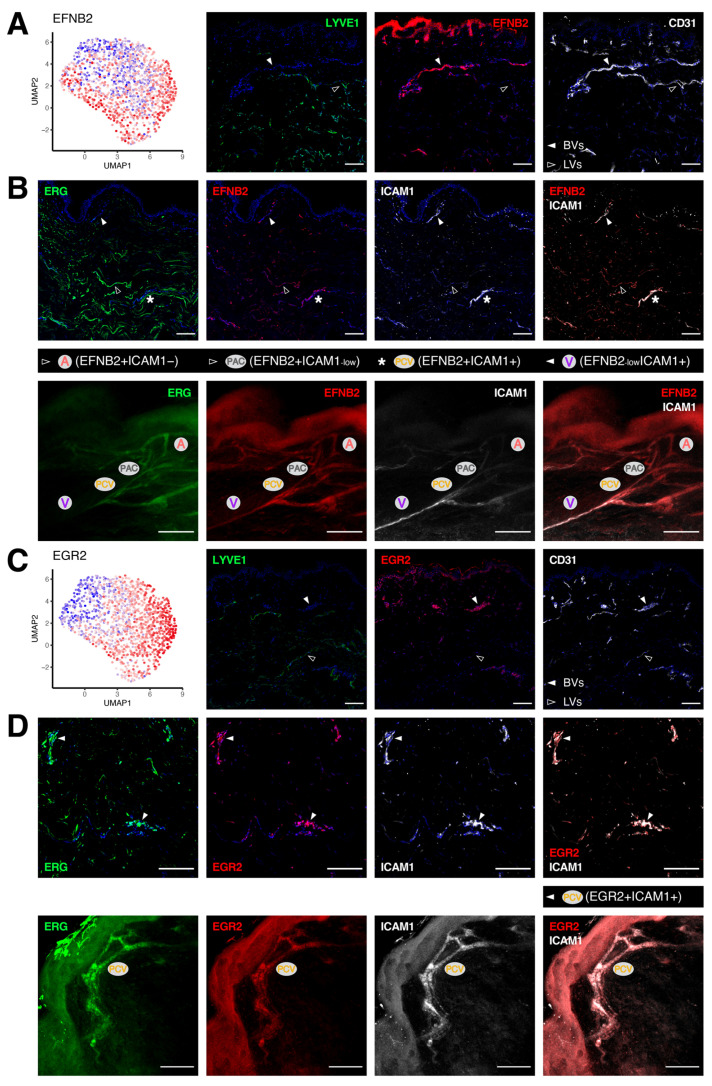
Gene signatures peculiar to the venular compartment. (**A**) EFNB2 expression measured by scRNA-seq (left) and immunofluorescence staining (right) denoting its abundance across different BEC subpopulations and its exclusive expression in human dermal BVs (white arrowheads). (**B**) Representative confocal images of skin sections (upper) and whole-mount skin blocks (lower) stained for ERG (green), EFNB2 (red) and ICAM1 (white). The unique composition of EFNB2 and ICAM1 expression enables the discrimination of arterioles (empty arrowheads), post-capillary venules (*) and venules (white arrowheads). (**C**) EGR2 expression is most enriched in the post-capillary venule cluster and is restricted to BVs (white arrowheads). (**D**) Representative section (upper) and whole-mount staining (lower) of ERG (green), EGR2 (red) and ICAM1 (white), highlighting EGR2+ICAM1+ BVs. Scale bars: 100 µm.

We next sought to dissect different BEC subtypes in the venular compartment. EFNB2 is used as a classic marker for arterial specification during development. Its expression was also observed in arterioles and capillaries (A/PAC/PVC clusters) in adult human skin (Figure 3A). In addition, *EFNB2* expression extended partly into the venular segments, residing at the rim of the PCV cluster. Since EFNB2 was more highly expressed in BVs (Figure 3A), we subsequently co-stained it with ICAM1 to compare their spatial localization in other healthy donors (Figure 3B). We were able to confirm the presence of different BEC subtypes both in skin sections and whole-mount staining (4/4 donors). In particular, the level of EFNB2 started to fade out when transiting from arterioles and capillaries deeper into the venules, whereafter its expression was superseded by ICAM1 at the convergence point. Their overlap as seen in the PVC and PCV clusters was only found at the junctions connecting pre-venular vessels to downstream venules. On the other hand, we identified the transcription factor early growth response 2 (EGR2) to exhibit a near-identical expression pattern as ICAM1 across all BV subtypes, with negligible expression in LVs (Figure 3C). While the abundance of EGR2 and ICAM1 was most prominent in the PCV cluster, their co-localization was evident in the immunofluorescence staining of skin sections and whole-mount skin blocks (4/4 donors, Figure 3D). In addition, we identified another marker, leucine rich alpha-2-glycoprotein 1 (LRG1), which was also exclusive to BVs and peculiarly enriched in the PCV cluster (Figure S3A,B). The presence of such subpopulation of BECs was again highlighted by BVs positive for both LRG1 and ICAM1 (2/3 donors, Figure S3C). ACKR1 expression was detected in all venular ECs (PCV/V/CV clusters), whereas the level of both SELP and EGR2 diminished in the CV cluster. To characterize better the venular ECs categorized as the CV cluster, we examined the expression of SELP and EGR2 in ACKR1+ BVs (Figure S3D,E). We were indeed able to confirm the identity of these BEC subtypes in SELP−ACKR1+ and EGR2−ACKR1+ BVs (3/3 donors).

### 3.4. Transcriptomic Signatures of Blood Vascular Endothelial Cells Constituting Arterioles and Capillaries

**Figure 4:**
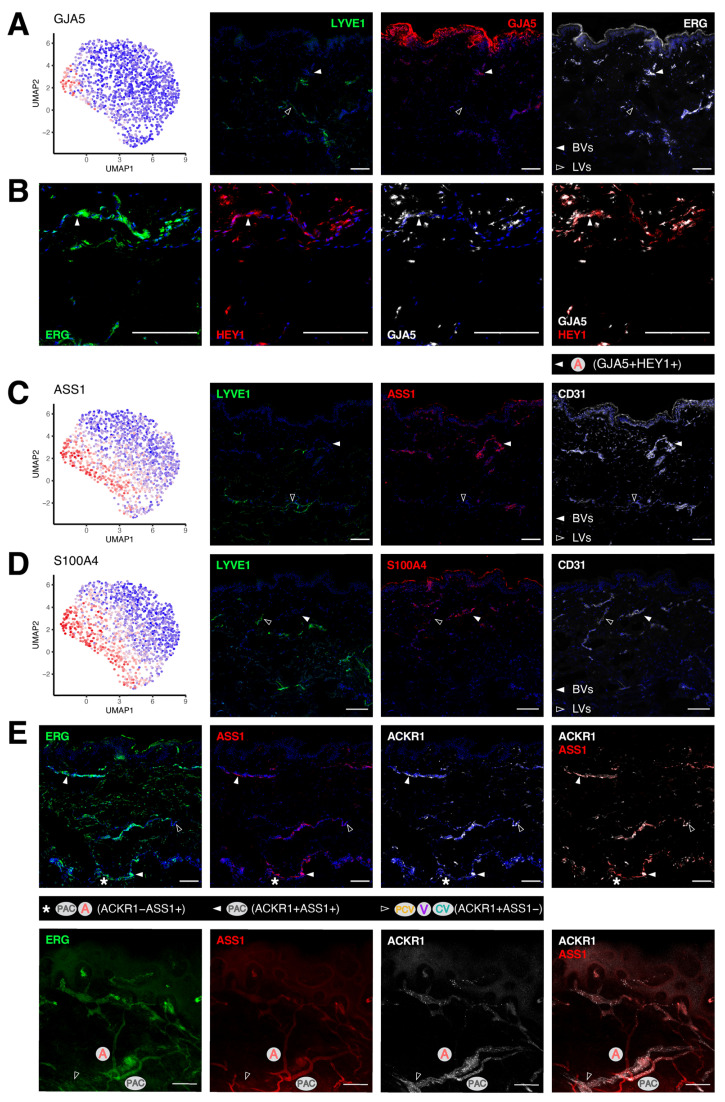
Molecular characterization of arteriole and post-arterial capillary endothelial cells. Exclusive expression of the arterial marker GJA5 by BVs (white arrowheads (**A**)) and its overlay with HEY1 (**B**). The expression of ASS1 (**C**) and S100A4 (**D**) was restricted to the A and PAC clusters (white arrowheads). (**E**) Differential localization of ACKR1 and ASS1 discerns arterioles and post-arterial capillaries. Representative confocal images of section (upper) and whole-mount staining (lower) of ERG (green), ASS1 (red) and ACKR1 (white). Scale bars: 100 µm.

Arterioles and capillaries differ from venules in terms of their transcriptomic patterns and biological functions (Figure 1C,D). While these BV segments were both highlighted by the expression of HEY1, a number of markers could be used to further separate arterioles from capillaries. For instance, the gap junction protein alpha 5 (GJA5) was most abundantly expressed in arterioles, but absent in LVs (Figure 4A). To corroborate the existence of this BEC subtype in human skin, we co-stained GJA5 with HEY1 in other healthy donors and spotted such a vessel type in a part of the HEY1+ segments (3/3 donors, Figure 4B). In comparison, argininosuccinate synthase 1 (ASS1) and S100 calcium binding protein A4 (S100A4) recapitulated to a large extent the expression pattern of HEY1, as well as its absence in LVs (Figure 4C,D and Figure S4A,B). *ASS1* expression merely intersected with *ACKR1* in the upper part of the PAC cluster, separating the arterioles and capillaries from the venular compartment. By conducting immunofluorescence staining on skin sections and whole-mount tissues, we gained a more complete view of the different BV subtypes based on their expression of ASS1 and ACKR1 (4/4 donors, Figure 4E), namely, the smaller ACKR1−ASS1+ arterioles, partially ACKR1+ASS1+ capillaries (the PAC cluster) and larger ACKR1+ASS1− venules (PCV/V/CV clusters). Likewise, *S100A4* overlapped with *ICAM1* only in part in the PAC cluster and we were able to locate S100A4+ICAM1− arterioles in human skin (3/3 donors, Figure S4C).

### 3.5. Molecular Characterization of Pre-Venular Capillary-Lining Endothelial Cells

**Figure 5:**
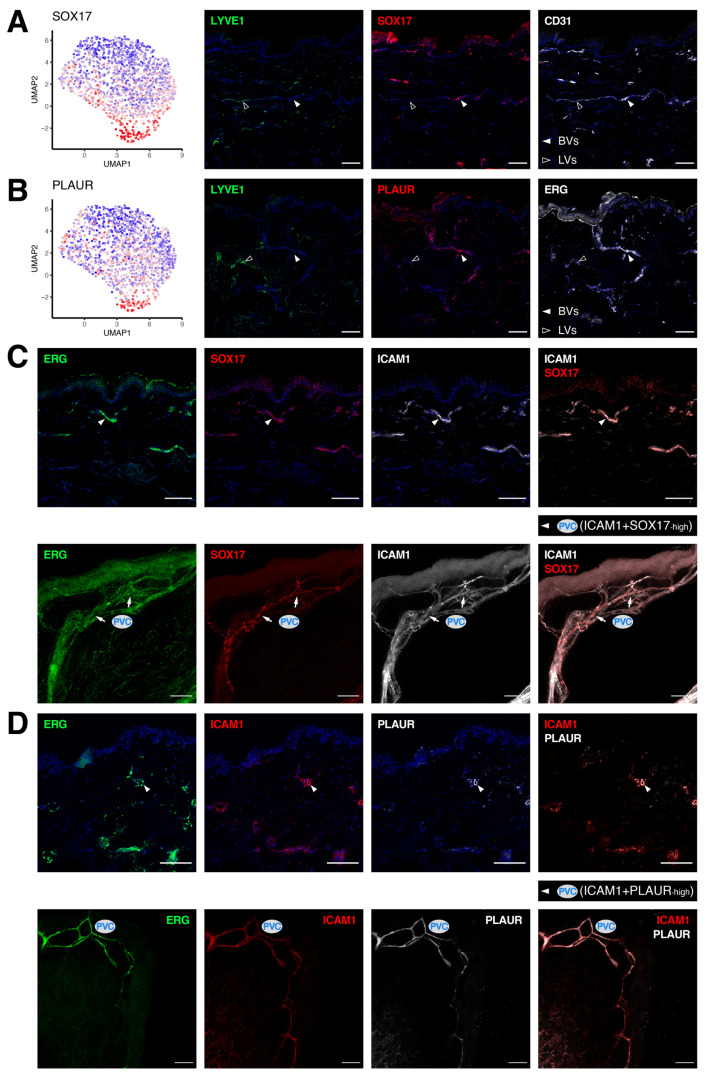
Pre-venular capillaries are pinpointed by novel markers. Specific localization of the PVC markers SOX17 (**A**) and PLAUR (**B**) in human dermal BVs (white arrowheads). (**C**) Intersection of ICAM1 (white) and SOX17 (red) highlights the segments of pre-venular capillaries. (**D**) Co-staining of ICAM1 (red) and PLAUR (white) in skin sections (upper) and whole-mount skin blocks (lower) denotes the pre-venular capillary cluster. Scale bars: 100 µm.

At the junction bridging the PAC cluster to the venular segments, we identified a unique cluster (PVC) of BECs not only expressing a multitude of venule-specific molecules, but many of the PAC signature genes (Figure 1C). Nonetheless, this cluster could also be distinguished by a collection of specific markers, including the SRY-box transcription factor 17 (*SOX17*) and urokinase plasminogen activator surface receptor (*PLAUR*; Figure 5A,B and Figure S5A). Of note, LVs were devoid of either SOX17 or PLAUR. Whilst the PVC subset is largely derived from one of the donors included in the scRNA-seq experiment (Figure S1B), we further validated our findings in multiple other donors using immunofluorescence staining. As ICAM1 expression spanned across a number of different BEC subpopulations, ranging from the PAC and PVC clusters all the way to the venular clusters (PCV/V), we thus employed it in co-staining to locate the segments of PVCs in skin tissues derived from other healthy donors. Intriguingly, SOX17+ vessels that were smaller in size were found at an intermediate position among the ICAM1+ segments, connecting the BVs coming from both ends (4/4 donors, Figure 5C). Similarly, PLAUR overlapped partially with ICAM1+ vessels, with its expression gradually declining towards the branches outside of the intermediate segments (4/4 donors, Figure 5D). Such structures likely represent the PVC segments bridging PACs and the venular compartment.

Overall, our findings delineate the transcriptional heterogeneity of different BEC subpopulations and provide a molecular mapping of individual BV compartments in healthy adult human skin (Table 1).

## 4. Discussion

The endothelia lining the lumen of arteries and veins exhibit substantial anatomical and functional differences. For instance, blood flow rates in arteries are much higher than in the venous circulation. Arterial and venous ECs thus encounter different levels of shear stress [33]. Moreover, their endothelia adopt a dissimilar organization of cell junctions to mediate intercellular adhesion and communication. While the junctions in arteries are much tighter than those in the venous vasculature, the latter manifests more loosely organized intercellular junctions to accommodate its functional requirements for leukocyte trafficking [33]. Benefiting from extensive embryonic studies on blood vascular endothelial lineage determination, an encyclopedic knowledge of the molecular specialization of vascular beds during arteriovenous differentiation has been acquired. In particular, the identities of arterial and venous ECs are marked by their differential expression of EFNB2 and its receptor EPHB4, even prior to the onset of blood circulation [36]. Since the phenotypic characteristics and functionality of distinct blood vascular compartments are finely maintained under homeostatic condition, we hereby exploited single-cell transcriptomic profiling of human dermal BVs to unveil different BEC subtypes present in adult skin and gradual changes in their marker expression patterns along the vascular tree.

Post-capillary venules represent the primary sites for leukocyte trafficking and subsequently the extravasation into peripheral tissues. The characterization of the atypical chemokine receptor ACKR1 has favored the identification of intact venous vasculature in multiple murine tissues [34]. In fact, ACKR1 also represents an important molecule that distinguished the downstream venular compartments from arterioles and post-arterial capillaries in human skin, in line with recent findings that EC junctional ACKR1 was required for efficient unidirectional transmigration of neutrophils from the venular lumen during inflammation [37]. A previous study, which investigated the distribution of EC markers in human dermal vascular beds via immunohistochemistry, has demonstrated that the arterioles, capillaries and venules present in the skin were all stained for CD31 and CD34, whereas VWF positivity was only detected in some of these BVs [38]. Congruently, VWF expression in our scRNA-seq data was predominantly found in the venular compartment, resembling the pattern of many endothelial adhesion molecules, including SELE, SELP and ICAM1. The expression of these adhesion molecules on the cell surface is crucial to the interactions between ECs and leukocytes [33]. The further upregulation of adhesion molecules can be elicited by pathophysiological stress and inflammatory stimuli, such as cytokines and chemokines, to assist the adhesion and trans-endothelial migration of immune cells [39].

While the transcriptomic signatures of venular ECs in the PCV and V clusters highlighted biological pathways associated with adaptive responses of ECs to stimulation, genes related to energy synthesis were more enriched in the CV cluster. This could be attributed to the fact that systemic veins carry blood lower in oxygen compared to arteries. Venous ECs further downstream consequently reside in a more hypoxic environment, which is an effective stimulus for ATP release from ECs [40]. We identified two additional markers that are specific to venular ECs, namely EGR2 and LRG1. The early growth response gene EGR2 is a transcription factor expressed in a variety of cell types (e.g., B and T cells) and is rapidly induced upon differentiation and antigen stimulation [41]. Markedly elevated expression of EGR2 was noted in skin and lung biopsies obtained from systemic sclerosis patients, particularly in BECs and fibroblasts [42]. Such induction of EGR2 in human dermal fibroblasts has been shown to be mediated by the TGFβ signaling pathway, yet the transcriptional functions of EGR2 in BVs at steady state or during inflammatory responses remain to be elucidated. On the other hand, recent studies have demonstrated that LRG1 greatly influences vascular growth, where it promotes proliferation, migration, tube formation and branching of human umbilical vein endothelial cells (HUVECs), via the modulation of TGFβ signaling [43,44].

Pre-venular ECs usually do not support leukocyte trafficking and indeed they manifested distinct transcriptomic profiles opposed to post-capillary venules. For example, ASS1 expression was more abundant in arterioles and post-arterial capillaries. While basal nitric oxide (NO) production in ECs may exert anti-inflammatory effects by inhibiting immune cell activation, it has previously been reported that endothelial ASS1 enhances NO production, which in turns attenuates the cell surface expression of vascular cell adhesion molecule 1 (VCAM1) and monocyte adhesion to ECs upon inflammatory stimuli [4,45]. Among the S100 family of calcium-binding proteins, S100A4 was also enriched in pre-venular ECs. Moreover, its expression intersected with our pre-venular capillary marker PLAUR (also known as uPAR), the receptor for urokinase plasminogen activator (uPA). uPA catalyzes the conversion of plasminogen to plasmin, which plays a fundamental role in extracellular matrix remodeling and angiogenesis [46]. Similarly, S100A4 can be released from ECs to stimulate the activation of plasminogen, which sequentially promotes the production of extracellular matrix degradation enzymes and the formation of capillary-like structures [47,48]. Moreover, not only is the plasminogen system important for extracellular matrix homeostasis, it also largely affects growth factor-induced endothelial cell proliferation and migration, rendering the pre-venular capillaries a potential hotspot for pathological angiogenesis [49,50].

The transcription factor SOX17 represents another marker that was enriched in pre-venular capillaries, which bridge post-arterial capillaries to downstream venules. SOX17 is known as a transcriptional regulator that orchestrates arterial specification, endothelial integrity and modulates tip cell differentiation during vessel sprouting [51,52,53]. In line with the co-expression of ICAM1 and SOX17 in the PVC cluster, it has recently been shown that the level of SOX17 was markedly elevated in ECs upon inflammation, which was essential to the induction of ICAM1 expression and monocyte adhesion [54]. Arterioles and post-arterial capillaries share a high level of molecular similarities. We corroborated the conserved expression of EFNB2 in arterioles and capillaries in adult human skin. Despite that, we identified markers (GJA4 and GJA5) that were exclusively expressed in human dermal arterioles, setting them apart from downstream vessel segments. On top of early studies showing the localization of the two gap junction proteins GJA4 and GJA5 (also known as connexins 37 and 40) in the arterial system in mammals, a more recent single-cell analysis of coronary artery development has nominated these two markers as mature artery-specific genes in late embryonic stages [55,56]. The induction of GJA4 and GJA5 in arteries relies on the NOTCH signaling pathway that is indispensable to the determination of arterial cell fate [57,58].

The differential transcriptomic landscape across distinct BEC subtypes underlies the disparity in their functions, which may also be ascribed to the regulatory network of upstream transcription factors. The pan-endothelial transcription factor ERG is a pivotal regulator of endothelial homeostasis from embryonic development throughout adulthood [35]. From our single-cell analysis, we also identified a list of cell type-specific transcription factors that are differentially expressed among the BEC clusters. For instance, forkhead box O1 (FOXO1), REL and interferon regulatory factor 1 (IRF1) were enriched in the cluster of post-capillary venules in a similar manner. IRF1 and REL, a member of the NF-κB family, are key mediators of VCAM1 expression during endothelial inflammation [59,60]. FOXO1, on the other hand, interacts with the promoter of endothelial nitric oxide synthase (eNOS) to repress its expression, which opposes the role of the pre-venular endothelial marker ASS1 in promoting NO production [61]. Some other transcriptional regulators, such as ETS2, nuclear factor of activated T-cells 2 (NFATC2 or NFAT1) and TGFβ-induced factor homeobox 1 (TGIF1), were more abundantly expressed in the pre-venular capillary cluster. While they have all been implicated in regulating EC inflammatory responses, NFATC2 in particular augments VCAM1 expression during inflammation [62,63,64]. In comparison, MECOM and ETS1 were more restricted to pre-venular vessels. In fact, ETS1 is required for EC survival and angiogenesis, whereas MECOM specifically marks mature embryonic arteries [56,65]. It is worth noting that comparative analyses of transcriptomes and proteomes often reveal a certain extent of concordance and incongruence, as cellular functions are also tightly orchestrated by multi-layered processes of post-translational modifications [66]. Future studies are thus required to validate and examine the biological relevance of different subtype-specific transcriptional regulators in adult human skin.

Two recent studies have provided new insights into the expression profiles that underpin the heterogeneity and organotypicity of healthy human cutaneous BECs [67,68]. In line with their findings, our study provides a systematic characterization of blood vascular endothelial heterogeneity in healthy adult human skin at single-cell resolution, featuring a detailed investigation of the spatial localization of seven novel subtype-specific vascular markers via extensive immunofluorescence co-staining with conventional markers. We also noted that human adult skin samples in general exhibit strong autofluorescence in the green channel (e.g., at the epidermis and extracellular matrix; Figure S6A–D), hence special caution should be taken to avoid misinterpretation and we hope that the markers presented in this study could facilitate the discernment of dermal vessel subtypes, especially in longer fluorescence wavelengths. Moreover, our findings could, to some extent, be used to identify different blood vessel subtypes in other organs. For instance, many of our BEC subtype-specific genes (Figure 1C), including the conventional markers CXCL12, ACKR1 and SELP as well as the novel markers GJA5, S100A4 and PLAUR, are also found to be differentially expressed in the respective cluster in human lung [69]. This indicates a certain level of conservation in BEC heterogeneity among different organs. These findings thereby provide an important resource for the vascular biology community and may lay the ground for future studies dissecting the differential activation or transformation of individual BV compartments under pathological conditions.

## Figures and Tables

**Table 1:** Summary of marker expression in different BEC subtypes.

Clusters	GJA5	ASS1	HEY1	S100A4	SOX17	PLAUR	EFNB2	ACKR1	ICAM1	EGR2	LRG1	SELP
**Arterioles (A)**	+	+	+	+	+	−	+	−	−	−	−	−
**Post-arterial capillaries (PAC)**	−	+	+	+	+	low	+	low	low	low	low	low
**Pre-venular capillaries (PVC)**	−	low	+	+	high	high	+	+	+	low	low	low
**Post-capillary venules (PCV)**	−	−	−	−	low	low	+	+	+	high	high	+
**Venules (V)**	−	−	−	−	−	−	low	+	+	low	low	+
**Collecting venules (CV)**	−	−	−	−	−	−	−	+	low	−	−	+

## Data Availability

The scRNA-seq data are available at ArrayExpress under the accession number E-MTAB-10137.

## Supplementary Materials

The following supporting information can be downloaded at: https://www.mdpi.com/article/10.3390/cells11071111/s1, Figure S1: scRNA-seq quality metrics and differentially expressed transcription factors; Figure S2: Expression profiles of conventional markers; Figure S3: Expression patterns of venular markers; Figure S4: Expression profiles of arteriole and capillary markers; Figure S5: Expression patterns of pre-venular capillary markers; Figure S6: Representative images of human skin tissues stained with isotype controls; Table S1: Clinical information of healthy skin samples used in the study; Table S2: Differentially expressed genes in each BEC cluster (log fold change ≥ 0.25 and adjusted *p* < 0.05).
