# Supplementary material for: Novel Blood Vascular Endothelial Subtype-Specific Markers in Human Skin Unearthed by Single-Cell Transcriptomic Profiling
Source: Cells. 2022 Mar 25;11(7):1111. doi: 10.3390/cells11071111 (PMC8997372; doi:10.3390/cells11071111)
Supplement: Supplementary file 1 [file cells-11-01111-s001.zip › cells-1640935-supplementary.pdf]

Supplementary Materials

Novel Blood Vascular Endothelial Subtype-Specific Markers in Human Skin Unearthed by Single-Cell Transcriptomic Profiling

Yuliang He [1,†], Carlotta Tacconi [1,2,†], Lothar C. Dieterich [1], Jihye Kim [1], Gaetana Restivo [3], Epameinondas Gousopoulos [4], Nicole Lindenblatt [4], Mitchell P. Levesque [3], Manfred Claassen [5,6] and Michael Detmar [1,*]

[1] Institute of Pharmaceutical Sciences, Swiss Federal Institute of Technology (ETH) Zürich, 8093 Zürich, Switzerland
[2] Department of Biosciences, University of Milan, 20133 Milan, Italy
[3] Department of Dermatology, University Hospital Zürich, 8091 Zürich, Switzerland
[4] Department of Plastic Surgery and Hand Surgery, University Hospital Zürich, 8091 Zürich, Switzerland
[5] Department of Internal Medicine I, University of Tübingen, 72074 Tübingen, Germany
[6] Department of Computer Science, University of Tübingen, 72074 Tübingen, Germany
* Correspondence: michael.detmar@pharma.ethz.ch
† These authors contributed equally to this work.

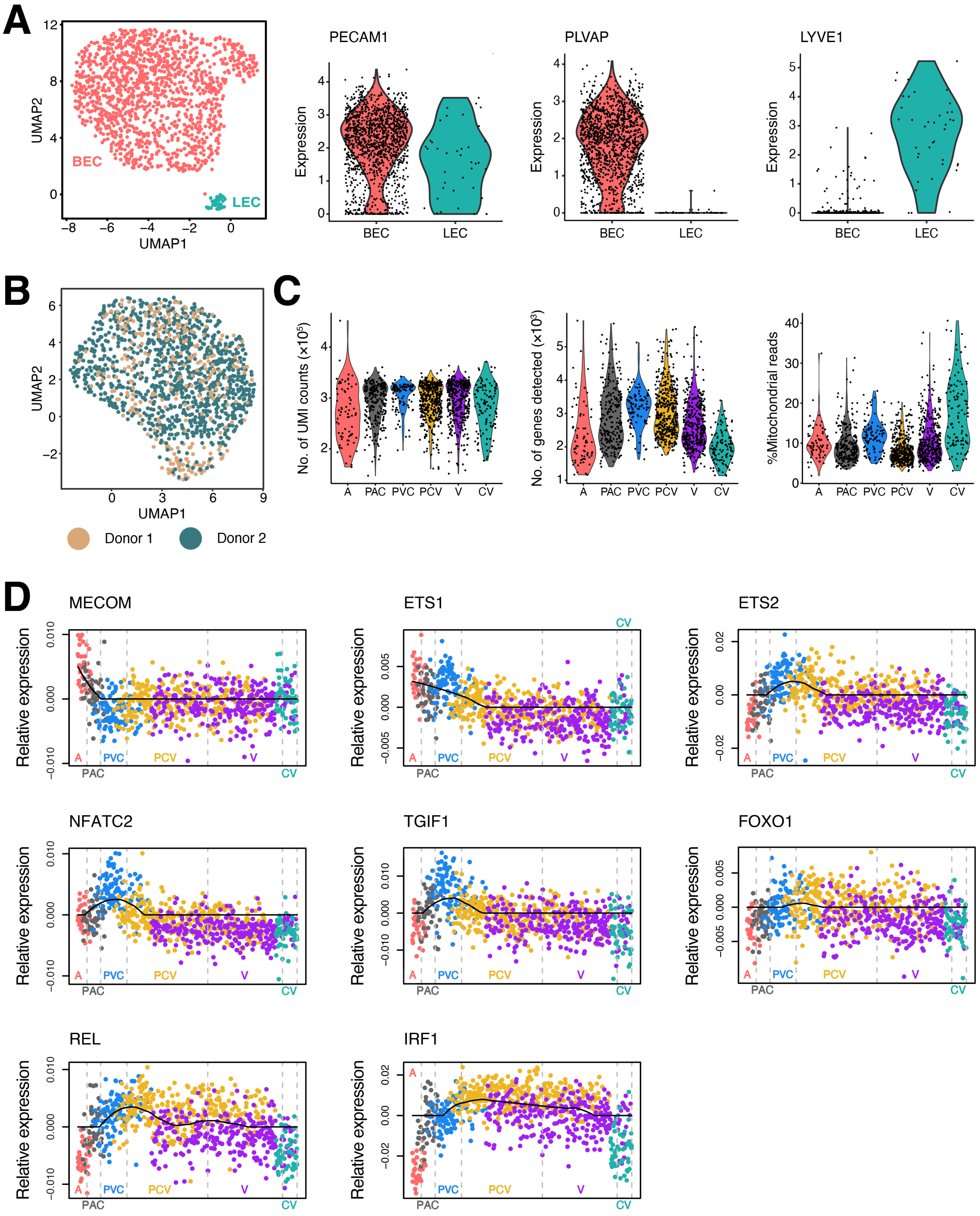

Figure S1. scRNA-seq quality metrics and differentially-expressed transcription factors. (A) Discrimination of blood vascular endothelial cells (BECs) and lymphatic endothelial cells (LECs) in accordance with the expression patterns of the pan-endothelial marker PECAM1, the dermal BEC marker PLVAP and LEC marker LYVE1. **(B)** UMAP plot of the BEC cluster color-coded by donors. **(C)** Violin plots showing the number of unique molecular identifier (UMI) counts, the number of genes detected and the mitochondrial contents measured by our scRNA-seq data. **(D)** Pseudo-temporal expression profiles of transcription factors differentially expressed in specific BEC subtypes. The lines indicate the average pseudo-temporal expression levels across the ordered cells.

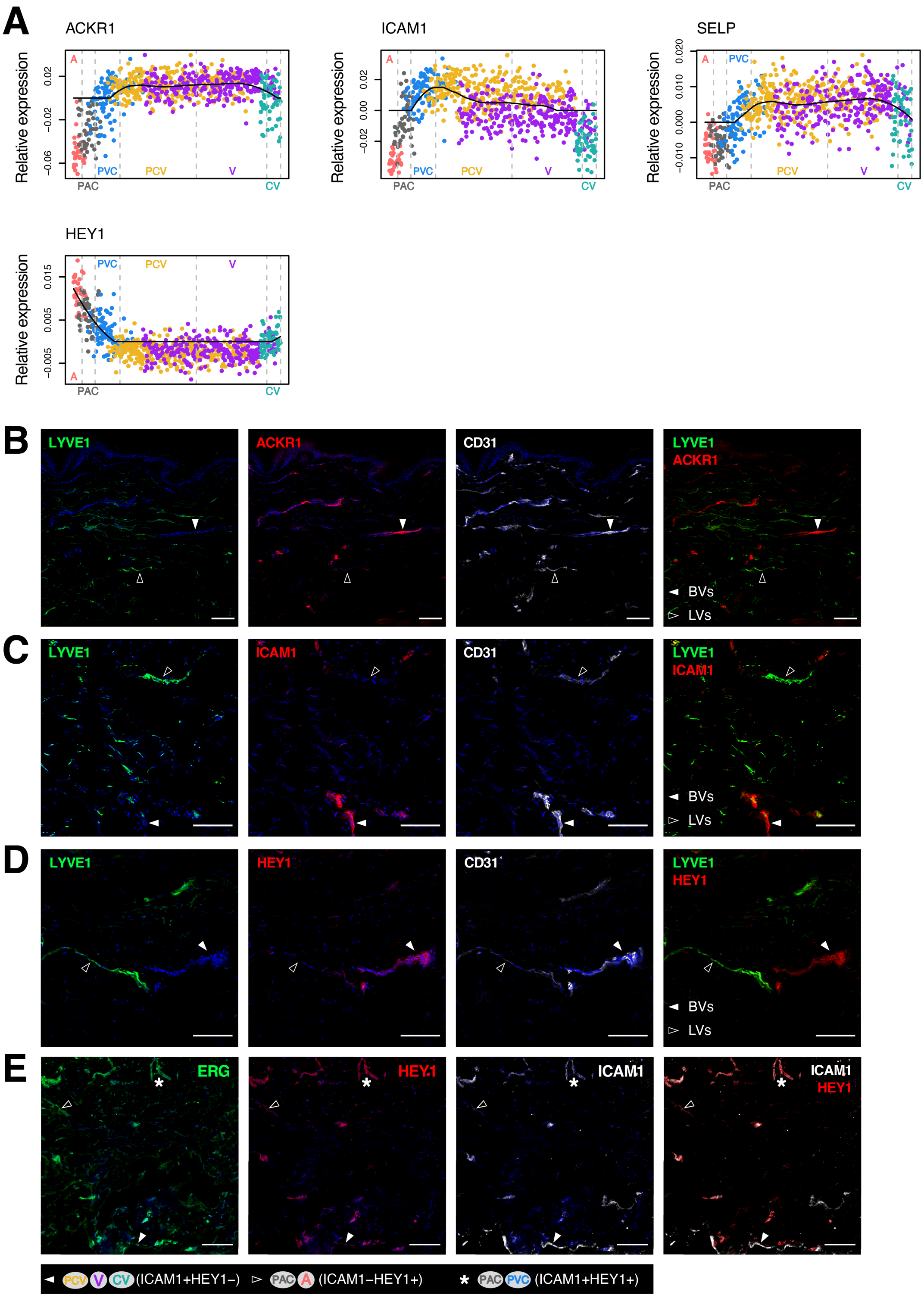

Figure S2. Expression profiles of conventional markers. (A) Pseudo-temporal expressions of ACKR1, SELP, ICAM1 and HEY1 across the ordered cells. Representative immunofluorescence images of skin sections stained for LYVE1 (green), CD31 (white) and ACKR1 (red; **B**), ICAM1 (red; **C**) or HEY1 (red; **D**), respectively. White arrowheads indicate CD31+LYVE1− blood vessels (BVs), while CD31+LYVE1+ lymphatic vessels (LVs) are denoted by empty arrowheads. **(E)** Co-staining of HEY1 (red) and ICAM1 (white) differentiates separate BV segments. Scale bars: 100 μm.

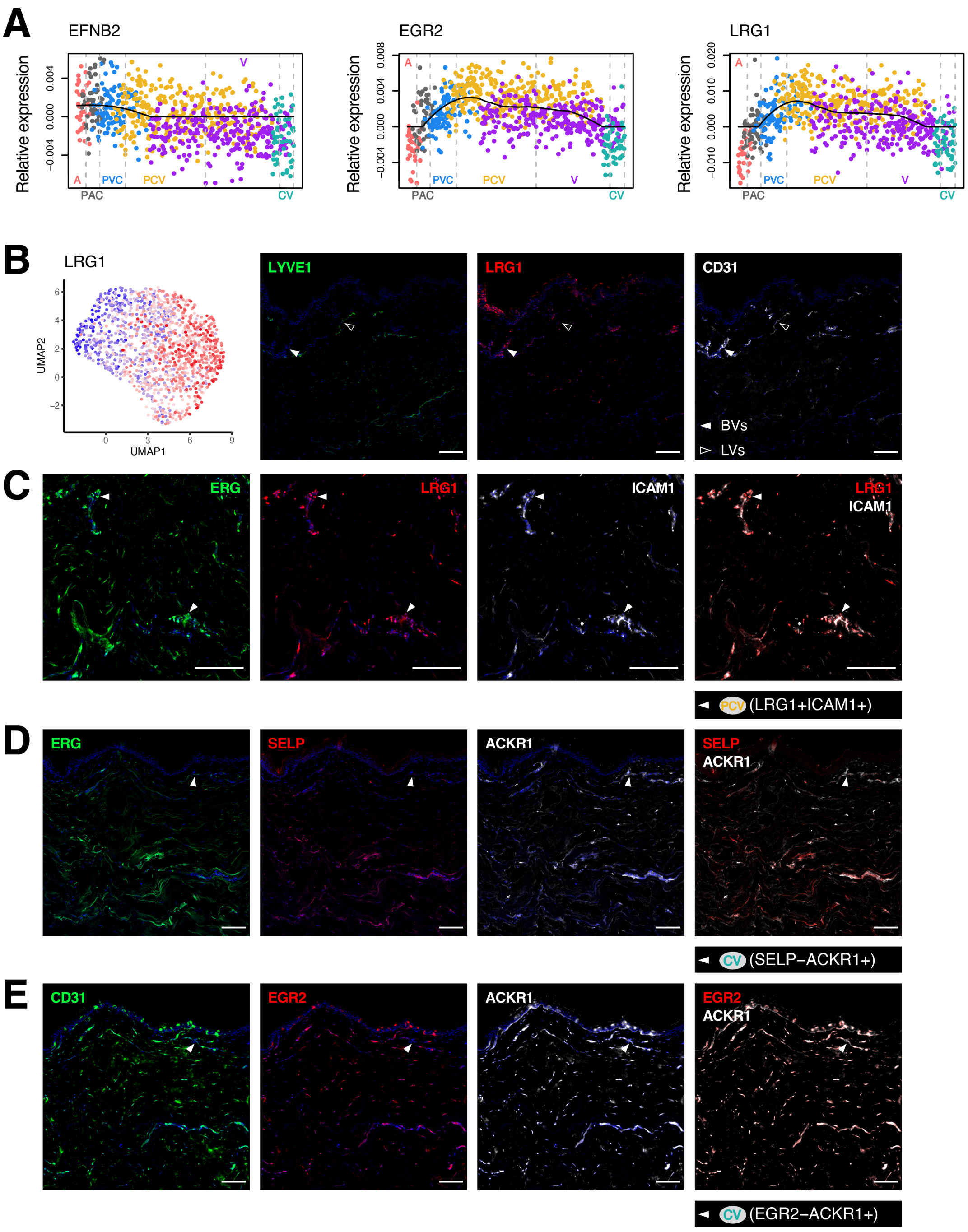

Figure S3. Expression patterns of venular markers. (A) Pseudo-temporal expression profiles of *EFNB2* and the post-capillary venule markers *EGR2* and *LRG1*. **(B)** Specific localization of the PCV marker LRG1 (red) in CD31+LYVE1− BVs (white arrowheads). **(C)** Co-staining of ERG (green), LRG1 (red) and ICAM1 (white) highlights the post-capillary venular ECs. The collecting venule cluster can be denoted by SELP−ACKR1+ (**D**) and EGR2−ACKR1+ (**E**) BVs. Scale bars: 100 µm.

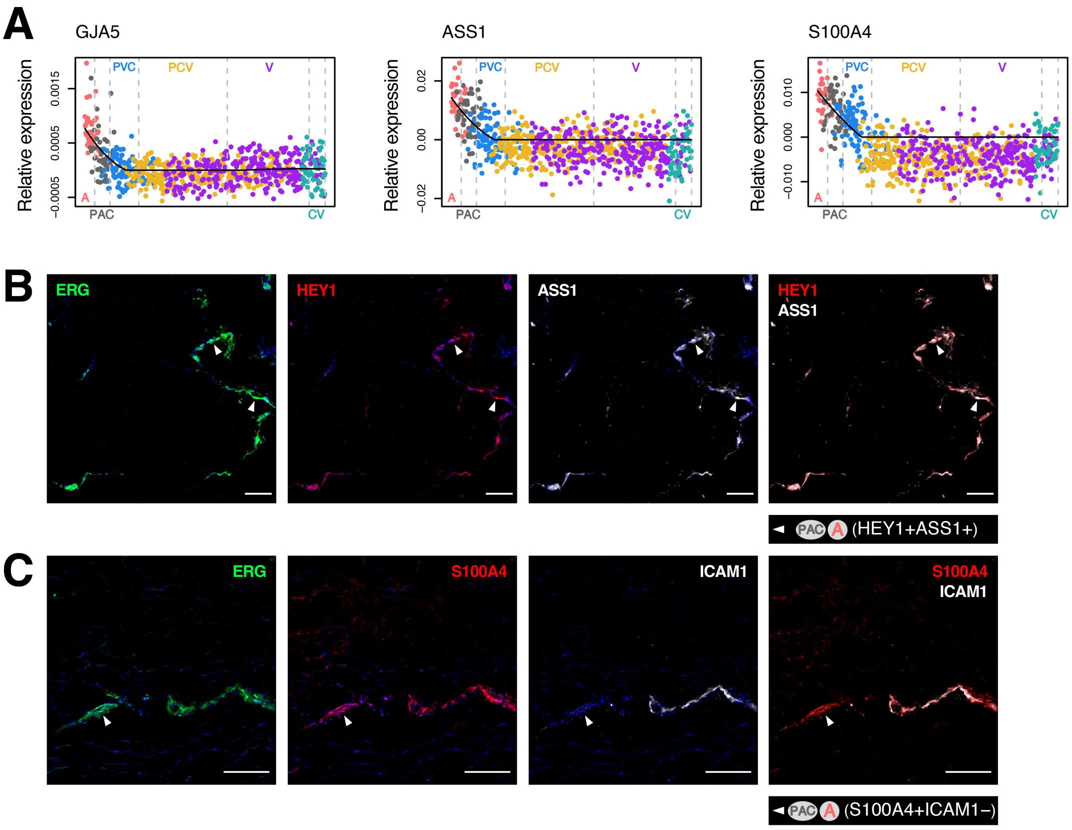

Figure S4. Expression profiles of arteriole and capillary markers. (A) Pseudo-temporal expression profiles of the arteriole and post-arterial capillary markers *GJA5*, *ASS1* and *S100A4* across the ordered cells. Distinguishable expression patterns of HEY1+ASS1+ (**B**) and S1004+ICAM1− (**C**) arterioles or post-arterial capillaries. Scale bars: 100 μm.

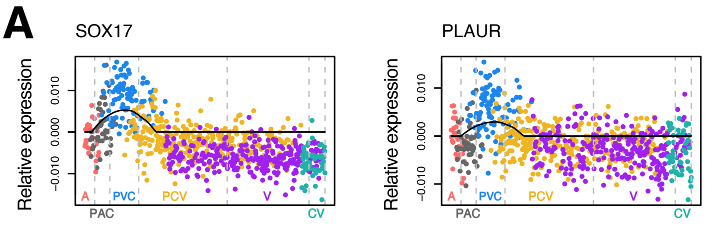

Figure S5. Expression patterns of pre-venular capillary markers. (A)

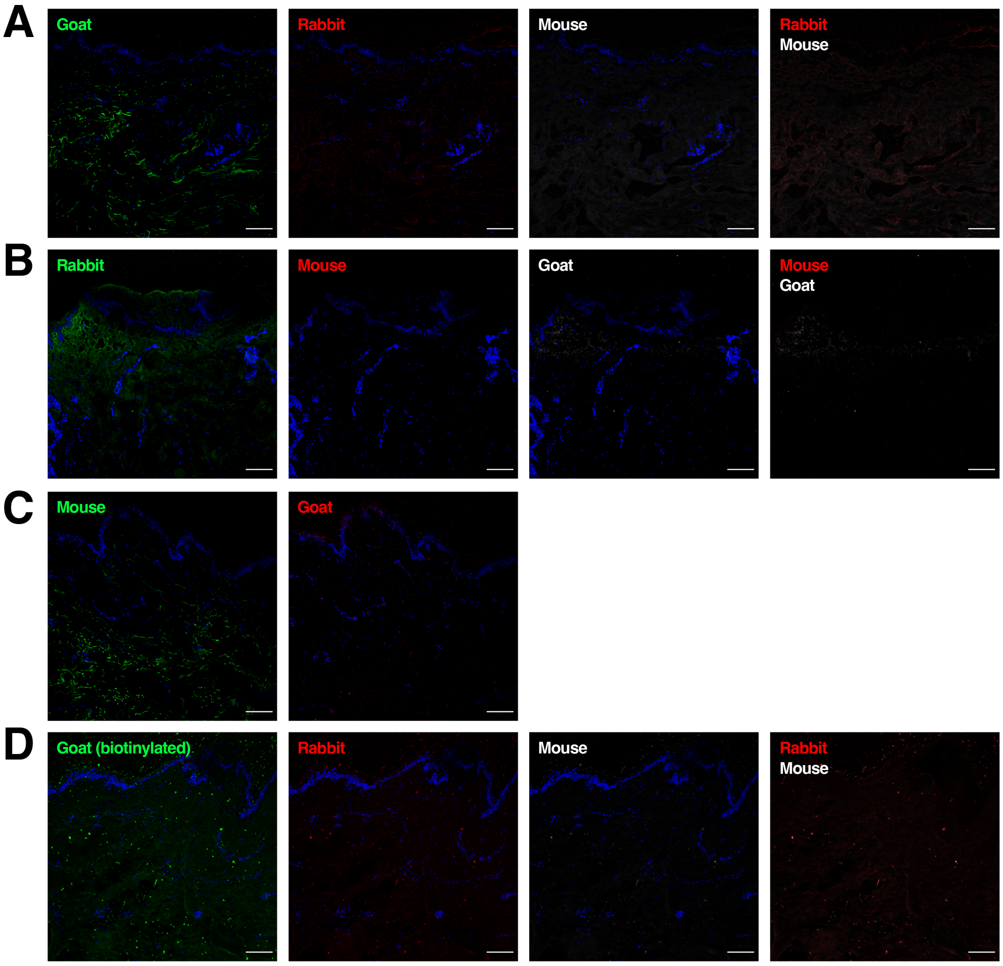

Figure S6. Representative images of human skin tissues stained with isotype controls (A-D). Scale bars: 100 μm.

Table S1: Clinical information of healthy skin samples used in the study

Donors	Sex	Age	Location	Surgery	Application
Donor 1	F	58	breast	plastic surgery	scRNA-seq
Donor 2	F	24	breast	plastic surgery	scRNA-seq
Donor 3	M	30	abdomen	plastic surgery	staining
Donor 4	F	41	abdomen	plastic surgery	staining
Donor 5	F	51	abdomen	plastic surgery	staining
Donor 6	F	30	breast	plastic surgery	staining
Donor 7	F	61	breast	plastic surgery	staining

Table S2: Differentially expressed genes in each BEC cluster (log fold change ≥ 0.25 and adjusted *p* < 0.05).

(1) Upregulated genes

<u>Arterioles</u>: *SLC9A3R2; SSUH2; HEY1; CXCL12; RGCC; SRGN; GLUL; ASS1; A2M; PODXL; SRP14; UTRN; PLPP1; KDR; RUNDC3B; FAM107A; CAV1; MMP2; EDNRB; ICAM2; ADGRF5; S100A4; FCN3; ALPL; NEBL; GJA4; TIMP3; LTBP4; PLS3; MECOM; JAG2; PLPP3; CFAP36; TEK; ABCB1; SLC12A2; RDX; RAMP2; PRR29; SEMA3G; GAS6; HEG1; AIF1L; PPA1; CAVIN3; CD9; ISG15; CDH5; SOS1; ARL15; ESAM; CAV2; BTNL9; LAPTM5; SLC6A6; EPAS1; PTPRG; CD109; NOTCH4; PLLP; C14orf37; CRIP2; TSPO; RHOC; IFI6; C8orf33; TM4SF18; AP1S2; LEPR; RAB31; NDUFA12; KYNU; IFI27; HECW2; MEF2A; ACE; COL4A2; NEMF; RPSA; ETS1; MTREX; LAPTM4A; Z93241.1; SRP9; AL603832.3; AL928654.3; FKBP9; CALM1; PSAP; APLP2; RMND5B; DEGS2; DAD1; ATP2A3; ARPC2; EEF1A1; PTMA; MGLL; RAMP2-AS1; ADAMTS6; SLC14A1*

<u>Post-arterial capillaries</u>: *SGK1; ASS1; S100A4; RGCC; ADM; RGS16; TCIM; TUBA1A; TSC22D1; IL32; RBP7; CRIP1; AL928654.3; TMEM204; NR4A1; RGS2; ID1; CD320; EFNA1; HES1; TIMP3; GGT5; ID2; CRIP2; AIF1L; DDIT4; PHYH; NOTCH4; KCNJ2; ADGRF5; ESAM; ID3; CAVIN3; SLC38A2; TOB1; CD9; HBEGF; FAM107A; C6orf48; TUBA1C; AQP3; PTP4A3; MGLL; CLEC14A; PODXL; LGALS1; SLC9A3R2; GSN; HEY1; IER2; SLCO2B1; CAV1; SPRY1; PLPP1; CDS2; ARL4D; OLFML2A; RCAN2; RAMP2; SERPINE1; KCNN3; GPRC5B; PTMA; CD99*

<u>Pre-venular capillaries</u>: *TM4SF1; MT2A; PNP; IL6; SOX17; FOSL1; G0S2; TXN; CSF3; EMP1; HMOX1; RCAN1; KLHL21; SOD2.1; AL355075.3; LITAF; PLAUR; ADAMTS4; AKAP13; STC1; ARPC5L; ARID5A; TMEM70; ELOC; SOD2; JMJD1C; SUSD6; NFATC2; AL049629.2; PDLIM1; STX11; ID1; LUZP1; TNFRSF12A; ADAMTS9; SPRY1; ID3; CD59; ARID5B; NOP16; MAP2K3; RPS16; TNFAIP3; GJA1; THBD; TOP1; TGIF1; CHIC2; SLC2A3; XBP1; ETS2; HIF1A; S100A10; SPAG9; NINJ1; MED24; SOX7; WWTR1; ZFAND5; EVA1C; ATF4; ELMSAN1; EDNRB; DUSP14; JAK1; BCAR3; ANXA1; DDX21; RPS29; CLEC2B; SAT1; VMP1; MT1X; TSC22D1; VAPA; TNFAIP2; RPS10; CYTOR; HIVEP2; TNFRSF10B; UPP1; CLIC1; BAZ1A; ICAM1; NOL4L; CYR61; S100A4; RPL35A; KLF6; TUBB6; USP12; PPA1; RPL37A; SPHK1; MT-ND3; EIF4E; GNL3; FOSL2; SOCS2; RPS10-NUDT3; CDKN1A; PLEKHO2; IFNGR2; WTAP; KDM6B; TXNL1; CHD7; RPS21; TMEM2; PMP22; HNRNPC; KBTBD2; RPL37; NDRG1; SHB; WDR43; TUBB4B; TP53BP2; SUB1; CYTH1; SNRPB; SLC38A2; UGCG; SPSB1; RALGDS; ELF1; RAB1A; MALAT1; NFKBIA; PFDN2; SQSTM1; RPL36AL; LMCD1; RPL24; ANGPT2; TRAF3IP2; RPS12; MLF1; SSH1; HES1; RSL1D1; NNMT; ZNF267; PPRC1; RPS17; PALM2-AKAP2; CWC25; NFE2L2; GADD45A; AKAP2; AC245033.1; H2AFZ; KAZN; MRPL32; CD200; ADGRG1; CYCS; SERTAD1; ARHGAP29; PCAT19; YBX3; SENP3-EIF4A1; ZNF385D; CSTB; PTMA; TUBA1C; RPL38; POLR1C; LDLR; BCCIP; BTG3; GRPEL1; CDC42EP2; AAED1; NME1; ITPRIP; RAN; MRFAP1; CD93; EIF4A1; RPS28; ILF2; RAB27A; BCAR1; EHD3; COL17A1; HAPLN3; EIF1B; BTF3; CDC42EP3; ZC3H15; RPL27; ENO1; HIPK3; SYNJ2; INSIG1; RPL21; SLC10A6; MSX1; RPLP2; PXDC1; TAGLN2; KCNJ15; SP100; CEBPG; AL162417.1; FUBP1; RPL36; PPP2R1B; KTN1; TRIM25; MIR22HG; RPS15A; LYAR; ETV6; UBE2N; RPS27; RPS24; RPL10A; RPS13; RPL32; CD55; PPFIBP1; RRBP1; TNFRSF6B; PSMD12; RPL30; NCL; RHEB; SNHG15; RPL19; NACA; UBE2D3; BACH1; S100A11; EHD1; RPL18; LDHA; RPL36A-HNRNPH2; TPT1; CNKSR3; MIDN; ICAM2; RPL35; RPL11; PHLDA1; RPS27A; EIF1; RPL26; RPL36A; S100A6; ABCF1; CLTB; ITPKC; KRT18; RAC1; JPT1; TMSB10; RPLP1; MYADM; FOXO1; PGS1; ADNP; RPL34; IRAK2; DLC1; EIF2S1; ARL4C; RTEL1-TNFRSF6B; NACC2; AK6; BCL6; SVIL; RPS7; NFATC1; UBE2D1; UTP3; IL6R; CDC42; NANS; TRIM16; BCL3; RPL17; RGCC; AC135178.2; MRNIP; RPL14; SLCO4A1; PRR29; PPTC7; PSMD11; CCT2; MLXIP; RPL17-C18orf32; SRSF4; COX17; RELA; RPS6; PPP2CA; RPS9; SNRPG; PLEKHB2; RAPGEF4; DCUN1D3; RPS8; DDX24; MARCH5; PIM1; UCK2; ATP5F1E; PKIG; C15orf39; MPRIP; MAST4; FAU; CRK; PER2; BIN1; HBEGF; SMAD7; SBNO2; CCNL1; BCOR; AC138811.2; HMGA1; TRIP10; RBM14-RBM4; MAFF; GOLGA4; RPL10; ARF4; STK17A; GNL2; HERPUD1; STC2*

<u>Post-capillary venules</u>: *ICAM1; NFKBIZ; MLKL; SELE; GLA; ZFP36; HSPD1; IRF1; CDKN1A; MAT2A; MED24; LRG1; RCAN1; HSPH1; EGR2; ATF3; BAG3; CSRP2; EGR1; JUNB; TIFA; FOS; RND1; BTG2; HSPA8; HSP90AB1; SOCS3; ADAMTS4; NAMPT; DDX5; GNAI3; SELP; HSPA5; DUSP1; TCP1; CCNL1;*

HOOK2; TNFRSF10D; DNAJA1; ITPKC; RAB5A; DNAJB1; SDCBP; CLK1; MIR22HG; ZC3H12A; PCF11; TNFAIP3; PPP1R15A; ENPP2; IL6; C2CD4B; INSIG1; NASP; DDX58; ACKR1; IFRD1; TSC22D3; MAP3K8; UGCG; C6orf62; MCL1; ZC3HAV1; LDLR; PMAIP1; STIP1; COQ10B; SERTAD1; WSB1; NFKBIA; DEDD2; HAPLN3; IL1R1; KLF10; CKS2; CLU; CTSC; HNRNPA2B1; TAP1; LDHA; EIF1; SEPHS2; PPP1R10; UBE2D3; IQCN; ZFAND5; RSRP1; CTGF; SLC1A5; SERPINH1; UBC; SLC25A25; GTF2H1; KMT2E; AC058791.1; AKAP12; ELMSAN1; GNS; PDK4; MEOX2; TRIB1; MAFF; TNFRSF10B; DDIT3; RLIM; AKIRIN1; TNPO3; ZBTB5; SERPINB1; ZNF267; MIR222HG; MFAP1; THBD; XBP1; KLF4; CCDC32; SRSF1; SERTAD3; CLEC1A; PAF1; AC116366.3; H2AFZ; DNAJB4; ARL5B; INPP1; PRCP; MRNIP; REL; NNMT; LRRC1; AHSA1; CHORDC1; CTNNAL1; YPEL2; CLIC2; SHC1; HSPB8; SRSF5; PLK2; JMJD1C; CNKSR3; PNN; PROX1; EIF3I; NEDD9; ARL6IP1; TGFBR3; TSPYL2; SOD2; NARF; HSPA1A; EMP1; GTF2B

Venules: *CLU; VWF; ACKR1; SNCG; TSPAN7; EDN1; AF241726.2; CCL23; CCL14; RAMP3; CCL15-CCL14; PRCP; DUSP23; TFPI; SELP; FAM213A; TNFSF10; SELENOW; MYL12A; CST3; GIMAP7; SELE*

Collecting venules: *NEMF; NKTR; ATP5PF; MT-ND1; MT-RNR2; MT-CO2; MT-ND4; MT-RNR1; MT-ATP8; ATP5ME; MT-CYB; MT-ATP6; MT-ND4L; MT-ND5; MT-ND2; NDUFC1; REX1BD; FAM133B; MALAT1; MT-CO3; PRKAR1A; IER5; WDR6; COX7A1; CARHSP1; ERICH1; FTX; BRI3; GFM1; SNX24; GOLIM4; MT-ND6; MT-TE; TRA2A; BTAF1; SRSF6; MRPS18C; MCTS1; PHYHIPL; SNRPD3; NDUFA1*

(2) Downregulated genes

Arterioles: *SELE; ACKR1; ZFP36; SOCS3; RND1; CDKN1A; ICAM1; IRF1; DNAJA1; RGS16; CCL14; CCL15-CCL14; FOS; CCL2; ATF3; G0S2; SERTAD1; BAG3; ADAMTS4; NFKBIA; IL6; HSPA6; CSRP2; GLA; DNAJB1; NFKBIZ; HSPH1; JUNB; CXCL2; RSRP1; TMEM70; HSPD1; PLIN2; CLU; UPP1; EGR1; NNMT; ZC3HAV1; SELP; TFPI; EIF1; TNFAIP3; SLC2A3; KLF10; IER2; HSP90AB1; PPP1R15A; TIFA; MAP3K8; DEDD2; ZNF385D; C2CD4B; MIR22HG; HSPA1A; NAMPT; YPEL2; ELMSAN1; HOOK2; CD14; SOD2; ITPKC; ZFAND2A; HSPA1B; DDIT3; SLC1A5; LRG1; BHLHE40; CCNL1; SNAI1; HSP90AA1; DUSP1; SNCG; CTNNAL1; CPVL; KLF6; BTG2; CLK1; HAPLN3; LITAF; MYC; SCARB1; SOD2.1; AC020916.1; KLF4; ZC3H12A; MATN2; OSER1; THBD; NANS; SPHK1; HSPE1; INSIG1; ATF4; HSPB1; TRIB1; NASP; CACYBP; NEAT1; MAFF; HSPA8; PAF1; CCL23; H3F3B; SERPINH1; NMB; LDHA; TNFRSF10D; SEPHS2; GADD45B; SLC38A2; EIF4A3; TPD52L1; SLC3A2; AHSA1; IL1R1; IER3; NR4A1; UGCG; TSPAN7; CSF3; XBP1; PDK4; HSPB8; MRPL18; PRDM1; DNTTIP2; MAP2K3; ETS2; SSH1; COQ10B; OLFM1; CSF2RB; ELOC; SERPINB1; NXT1; REL; CD55; EIF3I; CKS2; EMP1; SERTAD3; PMAIP1; IFRD1; RAB5A; FAM213A; AKAP12; PIM3; TNFRSF10B; TRIM25; AF241726.2; S100A10; DNAJB4; NFIL3; MAT2A; TPT1; PRCP; IER5; BCL6; IFI16; BIRC3; FOSB; MEOX1; ANXA1; C21orf91; NUPR1; SMAD1; ZFAND5; NCOA7; HSPE1-MOB4; FOSL1; GNS; PLEKHO2; CHD1; DNAJB9; MMRN1; NECTIN2; TRA2B; CDC42EP3; NR2F2; ARID5A; DUSP23; INPP1; ANKRD10; COTL1; CEBPD; CSRNP1; MRNIP; ACTN1; TESC; TMEM173; TOP1; JUN; ELF1; NDRG1; MT-ND3; PDLIM1; TCEAL9; MLF1; TUBA1B; VWF; SQSTM1; HLA-DQA2; SRSF2; NDEL1; ARL5B; SHISA2; DUSP5; UBB; PDIA5; ABCD4; FOSL2; PHLDA1; MCL1; GNAI3; LIMS1; FAM241A; MT-CO3; MEOX2; CHD2; SHC1; SDCBP; PTK2B; ADIRF; TSC22D2; CARD19; LRRC1; CSF1; EGLN3; PIM1; TPM3; NOP58; MAD2L1BP; LYRM1; TSPYL1; PCDH19; CLIC2; CST3; SOX4; SLC25A25; UBE2D3; LPCAT4; NOLC1; HLA-DQB2; ARIH1; TRIM14; HLA-DQA1; DDX3X; CYTL1; TRAPPC4; TCP1; SH3BP5; RALGDS; NOCT; NOL4L; DLC1; FCGRT; RAMP3; NFKBID; MT-CYB; HLA-DMB; CNN3; HNRNPH1; ERVK3-1; SEMA6A; TUBB4B; LMCD1; CLN8; AL162417.1; HLA-DRA; AC025259.3; PLEKHA7; SOCS2; CYTH1; MT-ND2; TNFRSF12A; MARCKSL1; AC020915.1; GMFG; AC020915.5; FBXO11; IRF8; PPRC1; ZFC3H1; USP36*

Post-arterial capillaries: *SELE; CLU; G0S2; ACKR1; SELP; IL6; VWF; CCL14; CCL15-CCL14; TPD52L1; PRCP; CCL23; CSRP2; ICAM1; SNCG; TFPI; FAM213A; TSPAN7; HAPLN3; OLFM1; NNMT; ZNF385D; LRRC1; CTSC; IL1R1; LIFR; AF241726.2; EVA1C; C2CD4B; MMRN1; PLCB4; CSF2RB; NRN1; LPCAT4; SNCA; MYL12A; TMSB4X; IL33; PERP; NPC2; ADIRF*

Pre-venular capillaries: *HSPA6; DNAJB1; HSPA1A; HSPH1; HSPA1B; TXNIP; DUSP1; FOS; DEPP1; PRCP; PLK2; GIMAP7; EDN1; HSPD1; PDK4; CACYBP; EGFL7; ZC3HAV1; HSP90AA1; HSPA8; CLU; HSP90AB1; HYAL2; DDIT4; A2M; GSN; VWF; PECAM1; TSC22D3; JUN; MRPL18; ZFAND2A; DEDD2; EIF4A2; GIMAP4; HSPB1; PLVAP; DNAJB4; TSPAN7; AHSA1; JUNB; LIFR; TSPAN4; GLUL; TACC1; NOSTRIN; MFNG; RAMP3; TNFSF10; CLEC14A; HOOK2; CTSC; CCL23; NFKBIZ; ZFP36; FCGRT; CLDN5; ZFYVE21; PSAP; ARL6IP1; CXorf36; HSPE1; AKR1A1; UBC; RAPGEF3; IGFBP4; AC004922.1; FAM213A; AF241726.2; SYNGR2; SNCG; RNASE1; TSPO; SNAP23; SRSF3; ARPC1B; SERTAD3; PPP1R15A; UBB; RGS2; SLC29A1; DDIT3; VAMP3; MT-ND4; FIS1; PHYH; COA3; GUK1; AC004691.2; CAVIN3; HSPG2; GIMAP1; STMN1; HLA-DRB5; CCDC85B; IRF1; SEPHS2; FKBP9; FKBP1A; MMACHC; JAM2; UBE2L6; MYL12A; BCAP31; NOSIP; RN7SL832P*

Post-capillary venules: *RGCC; ASS1; S100A4; SLC9A3R2; RBP7; HEY1; ID1; TCIM; SRP14; AL928654.3; CAV1; ADGRF5; MT-RNR2; CRIP1; MT-RNR1; SH3BGRL3; CXCL12; RAMP2; MT-ND5; CRIP2; MT-CO2; SPRY1; MALAT1; HES1; DAD1; PTMA; S100A6; CD99; GNG11; CSTB; ATP5PF; S100A11; IFITM3; HINT1; UQCRQ*

Venules: *RCAN1; TM4SF1; MT2A; RGCC; MED24; TSC22D1; S100A4; PNP; ASS1; CDKN1A; HEY1; ICAM1; SGK1; SOX17; EMP1; HSPA5; SLC9A3R2; PODXL; JMJD1C; ARID5A; AKAP13; THBD; MAT2A; SSH1; KDR; FOSL1; ELMSAN1; MAP2K3; TCIM; ZFAND5; LDLR; C6orf62; MRNIP; ADGRF5; ELOC; SQSTM1; DDX5; AL355075.3; AMD1; CRIP1; EGR1; AL928654.3; TUBB4B; MSN; LUZP1; PCF11; ADGRG1; PLK3; HERPUD1; SYNE2; KBTBD2; TMEM2*

Collecting venules: *ICAM1; CDKN1A; RCAN1; BAG3; GLA; NFKBIZ; HSPD1; ZFP36; ATF3; TM4SF1; DNAJA1; SERTAD1; PNP; RGS16; TMEM70; RND1; CLK1; DNAJB1; EGR1; RSRP1; ADAMTS4; XBP1; EIF4A3; JUNB; HSP90AB1; TUBB4B; ELMSAN1; EIF1; TNFAIP3; HERPUD1; DUSP1; UBC; PPP1R15A; NFKBIA; MIR22HG; IRF1; CYR61; GADD45B; LITAF; SLC38A2; INSIG1; TIFA; HBEGF; ITPKC; ZC3H12A; NEU1; FOS; LRG1; STOM; PPP2CA; CSRP2; HNRNPF; HOOK2; UBE2D3; CTGF; TUBA1B; EIF3I; CKS2; LDHA; UAP1; PSMB8; SERPINH1; LDLR; MAFF; PLK2; MCL1; SOD2; HSPH1; ZNF622; PPP1R10; C6orf62; THBD; DNAJB9; SENP3-EIF4A1; TCP1; STIP1; CCNL1; RAB5A; HSPA8; SLC3A2; SOCS3; PGK1; H3F3B; BTG2; NEDD9; FOSL1; PAF1; GJA1; OSER1; MAGED2; DEPP1; MRPL18; AC020916.1; PMAIP1; EGR2; GTF2B; HILPDA; ANXA1; IER2; DDIT3; SOD2.1; CACYBP; DDX58; ARID5A; ZFAND5; ARF4; UBB; WSB1; TAF7; H2AFZ; SLC2A3; TAP1; SERINC3; ATF4; NMB; KLF10; CHMP1B; HSPA2; AC058791.1; NINJ1; EFNA1; EIF4A1; INPP1; AL355075.3; CYCS; SQSTM1; AMD1; ILF2; NAMPT; PDLIM1; STX11; SLC1A5; AHSA1; BTG3; PHLDA1; ZFAND2A; TSG101; COPS3; ACTB; HAPLN3; NGRN; PSMD13; FOSL2; RAN; TXNL1; BRD2; GNAI3; FLOT1; MAT2A; NANS; DDX5; HNRNPH2; UGCG; ARRDC3; DLC1; TMEM173; TSC22D1; ZC3HAV1; TIPARP; C1orf43; BCL6; SOX17; COQ10B; SLC25A44; NR4A1; CHORDC1; GHITM; OAZ1; YWHAQ; TOX4; SLU7; MAP2K3; CD40; MLF2; NXT1; HSPA5; TUBA1C; CAV2; RBM14-RBM4; PSMD6; RBM4; JMJD1C; SUMF2; ADAMTS1; NNMT; TOB1; CHIC2; SP100; NGDN; PCF11; BCL10; MYC; DEDD; CHSY1; TMBIM1; SLC25A25; EIF5; OSTF1; ADRB2; AC091167.2; RHEB; TRIB1; TAGLN2; NOP58; KLF4; MAP1LC3B; ZNF644; SNRPB; PMP22; KMT2E; ERVK3-1; DCAF13; NECTIN2; UTP11; RAB29; HNRNPH3; ANP32E; NUFIP2; SSR2; PPP2R1B; SPARC; HOXD1; CRTC2; TNFRSF10D; PLLP; DNAJB4; NFIL3; HSP90AA1; JMJD6; SSH1; SERP1; VMP1; DDX39A; MARS; ALDOA.1; ALDOA; PLEKHO2; NXF1; DEDD2; FUS; TUBB; MARCH6; ZNF574; AC116366.3; SDCBP; DUSP5; PPP1R15B; ADAMTS9; PPRC1; PAFAH1B2; PSMC6; TPM3; EIF4A2; ARIH1; DNAJB6; AKIRIN1; EIF4B; FKBP4; AP2M1; IFI16; ESAM; ATP5F1B; ACAT2; BCAS2; TXLNA; WTAP; MYADM; TGIF2; MRNIP; NR4A2; AC020915.5; SLC41A1; SOX7; LUZP1; SAR1A; C2CD4B; GAPDH; IER3; RFC2; PSMB1; SLC39A1; EIF5A; EMC4; AC020915.1; ARL6IP1; PNRC2; RNF5; CAPZA2; ALPK1; AL049629.2; PSAP; DDOST; CD9; ARL2BP; MYNN; MIR222HG; NFE2L2; RTN3; NR1H2; RELB; CLIC1; MORF4L2; CDK2*
